# Supplementary material for: Genomic characterization of a diazotrophic microbiota associated with maize aerial root mucilage
Source: PLoS One. 2020 Sep 28;15(9):e0239677. doi: 10.1371/journal.pone.0239677 (PMC7521700; doi:10.1371/journal.pone.0239677)
Supplement: S1 Data — (PDF) [file pone.0239677.s003.pdf]

Supporting Information – Figures

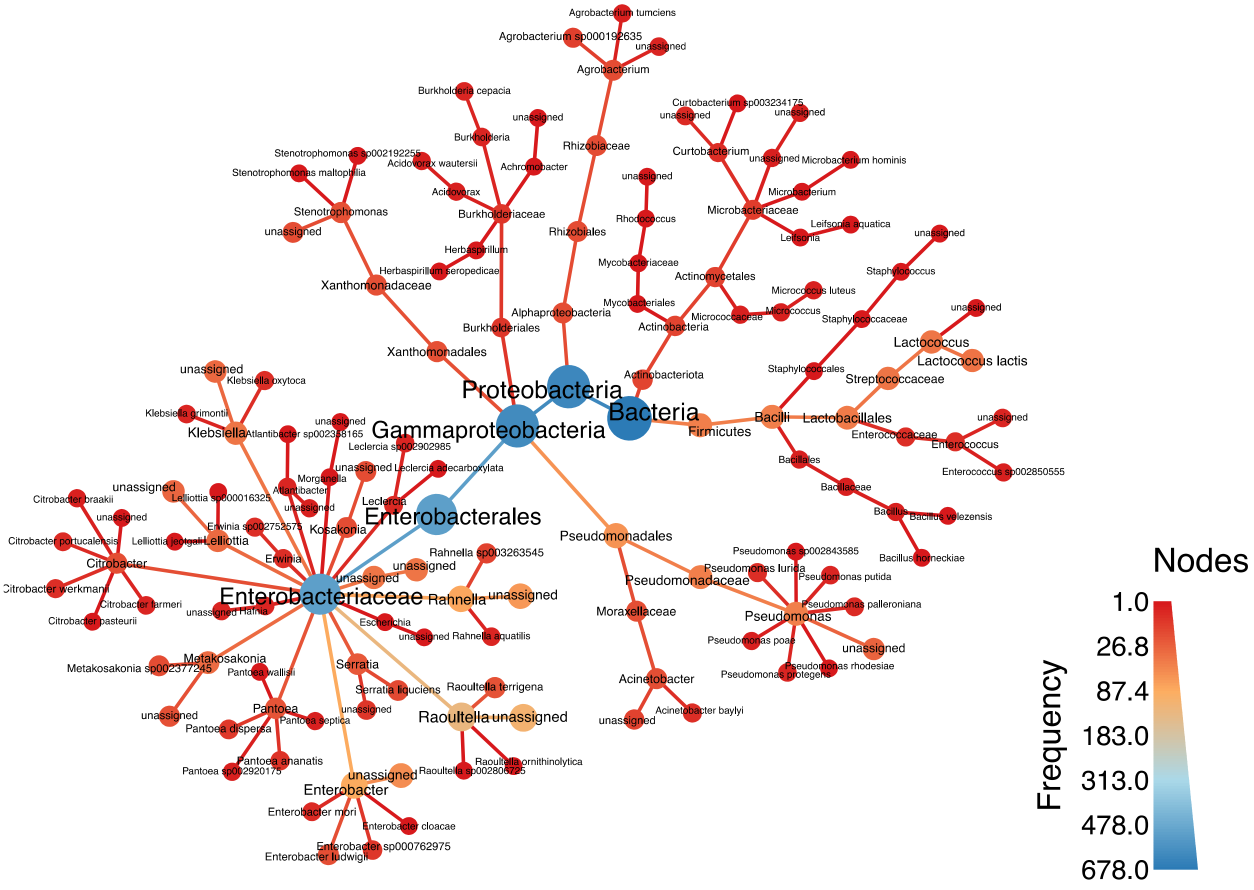

S1 Fig. Taxonomic classification of genome bins from mucilage isolates. Heat Tree showcasing the phylogenetic diversity of all Metabat [1] genome bins derived from isolate draft genome assemblies. Node size corresponds to the frequency of occurrence at each level of taxonomic classification. Dark blue corresponds to the highest observation count of a taxonomic assignment and dark red indicates low observation frequency.

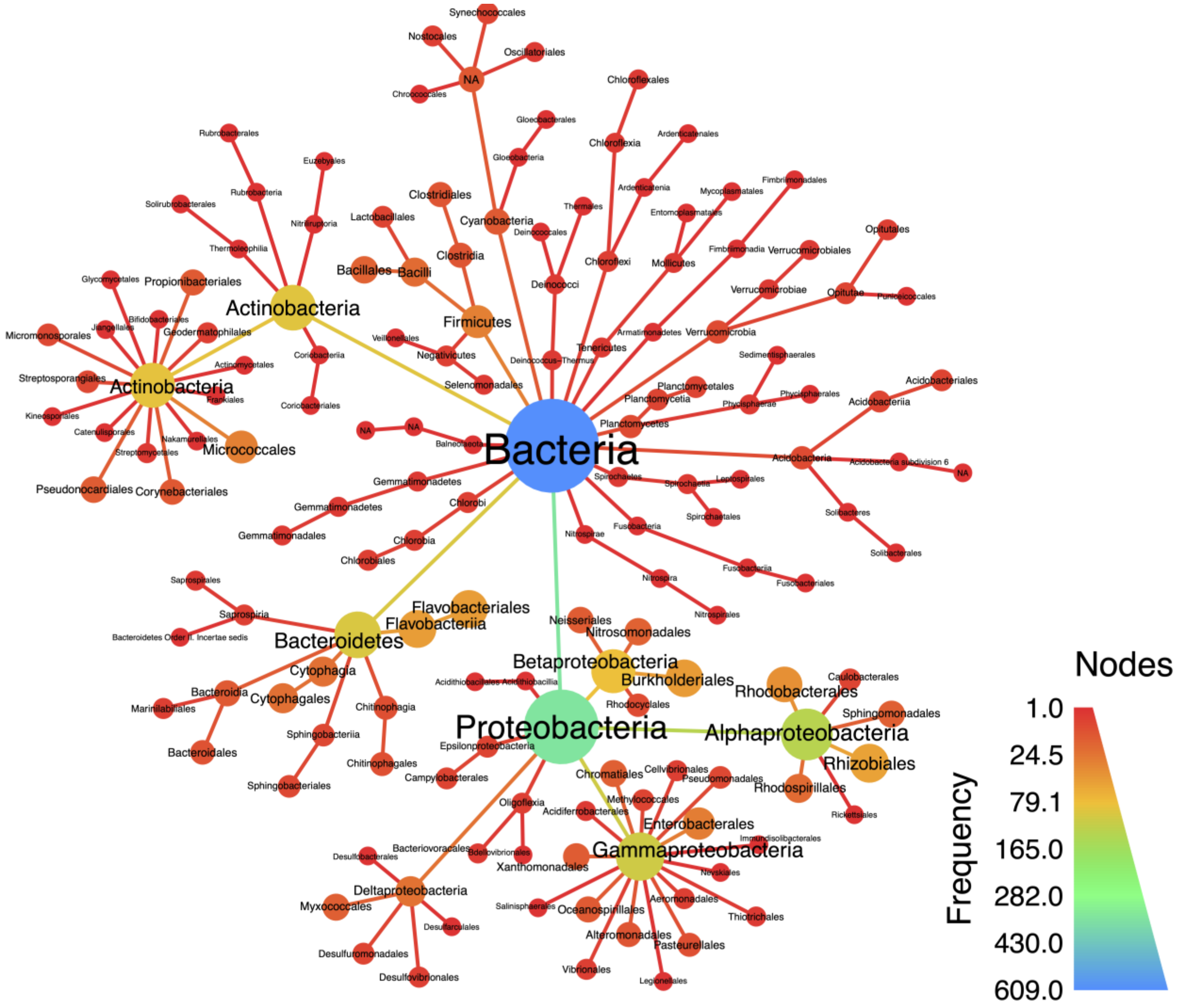

S2 Fig. Observation counts of classified taxa in the mucilage metagenome. Quality trimmed short reads of the OLMM00 mucilage metagenome were input to Kraken2 [2] and classified using the RefSeq complete database for microbial genomes installed with built-in commands of Kraken2. Bracken2 [3] was used to re-estimate classified read counts and the data was imported to R in biom-format. Taxa with readcounts less than 500 were filtered using Phyloseq [4] and the data were visualized using MetacodeR [5]. Terminal nodes represent taxa classified at the order level and node size corresponds to frequency of observation for each taxonomic level.

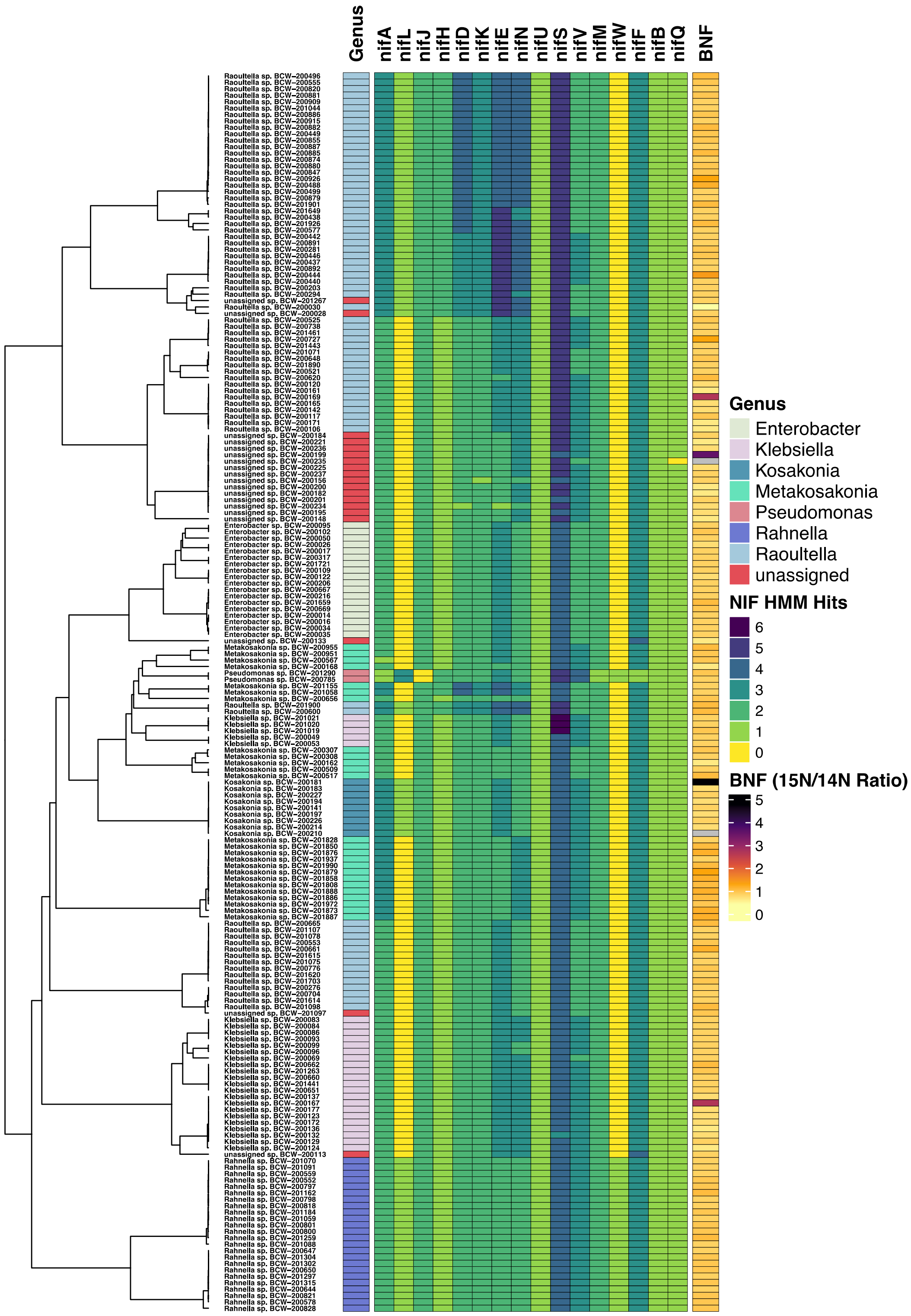

S3 Fig. *Nif* gene profile and BNF assay performance of Dos Santos Positive isolates. *Nif* gene profiles for isolate genomes of the DSP group were extracted and visualized independently

using the ComplexHeatmaps [6] package in R. Isolate genomes were clustered using the dendrogram output from Sourmash [7] and each genome row is presented along with annotations to indicate lowest common ancestor (LCA) classification data at the genus level.

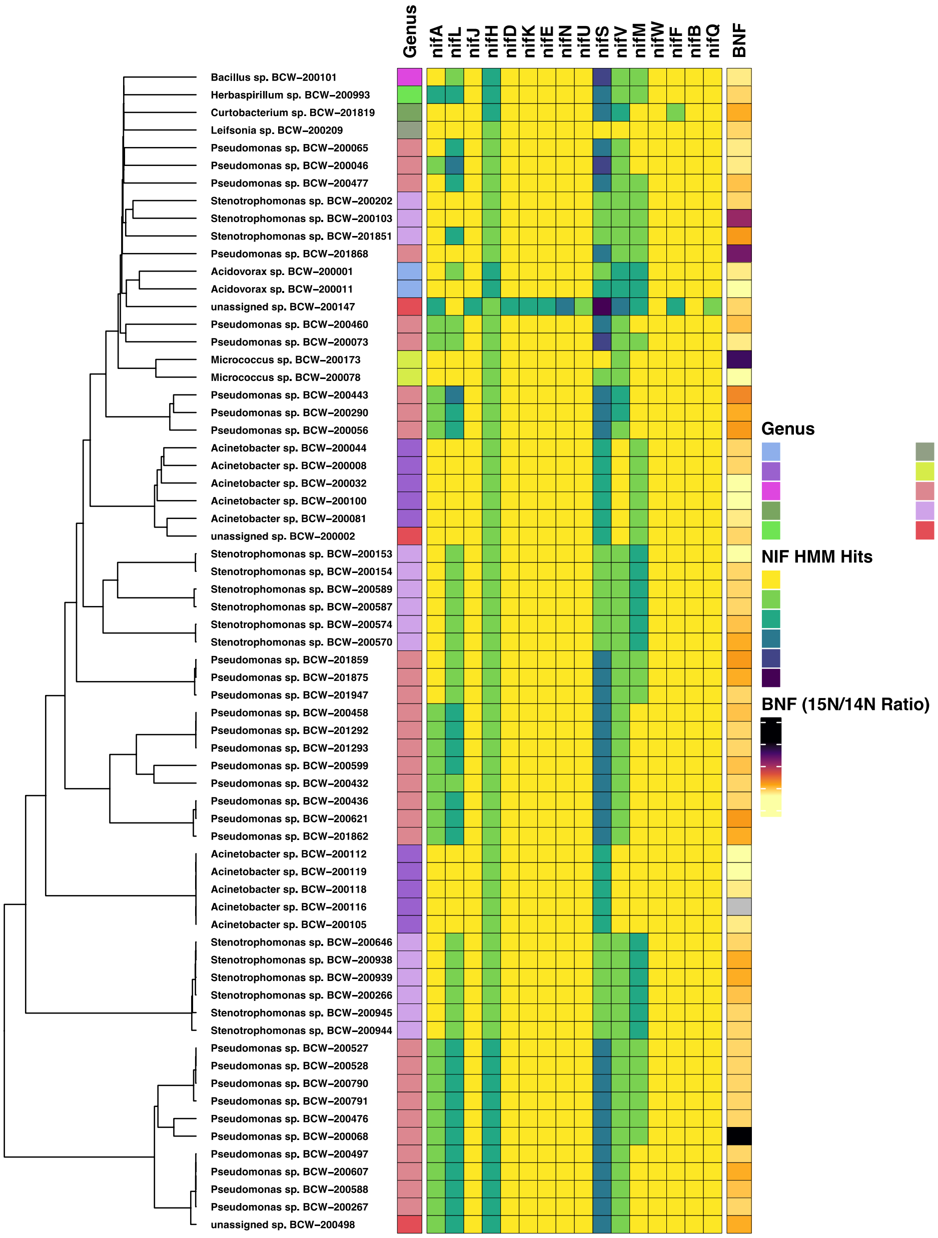

S4 Fig. *Nif* gene profile and BNF assay performance of Semi-Dos Santos isolates. *Nif* gene profiles for isolate genomes of the SDS group were extracted and visualized independently using the ComplexHeatmaps [6] package in R. Isolate genomes were clustered using the dendrogram output from Sourmash [7] and each genome row is presented along with annotations to indicate LCA classification data at the genus level.

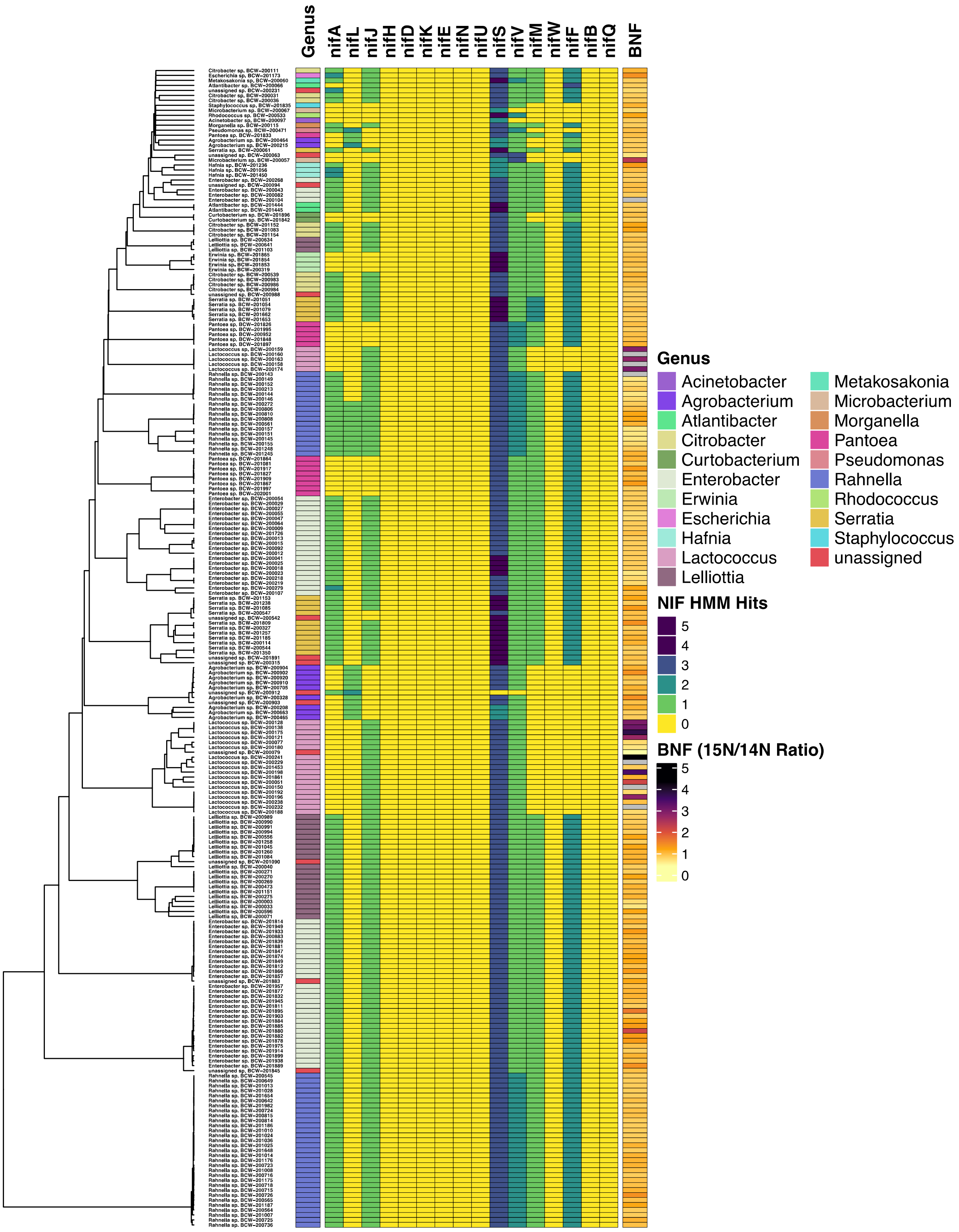

S5 Fig. ***Nif*** **gene profile and BNF assay performance of Dos Santos Negative isolates.** *Nif* gene profiles for isolate genomes of the DSN group were extracted and visualized independently

using the ComplexHeatmaps [6] package in R. Isolate genomes were clustered using the dendrogram output from Sourmash [7] and each genome row is presented along with annotations to indicate LCA classification data at the genus level.

References

1. Kang DD, Froula J, Egan R, Wang Z. MetaBAT, an efficient tool for accurately reconstructing single genomes from complex microbial communities. PeerJ. 2015;3:e1165. Epub 2015/09/04. doi: 10.7717/peerj.1165. PubMed PMID: 26336640; PubMed Central PMCID: PMCPMC4556158.

2. Wood DE, Salzberg SL. Kraken: ultrafast metagenomic sequence classification using exact alignments. Genome Biol. 2014;15(3):R46. Epub 2014/03/04. doi: 10.1186/gb-2014-15-3-r46. PubMed PMID: 24580807; PubMed Central PMCID: PMCPMC4053813.

3. Lu J, Breitwieser FP, Thielen P, Salzberg SL. Bracken: estimating species abundance in metagenomics data. Peerj Computer Science. 2017;3:e104. doi: ARTN e104

10.7717/peerj-cs.104. PubMed PMID: WOS:000425411300002.

4. McMurdie PJ, Holmes S. phyloseq: an R package for reproducible interactive analysis and graphics of microbiome census data. PLoS One. 2013;8(4):e61217. Epub 2013/05/01. doi: 10.1371/journal.pone.0061217. PubMed PMID: 23630581; PubMed Central PMCID: PMCPMC3632530.

5. Foster ZS, Sharpton TJ, Grunwald NJ. Metacoder: An R package for visualization and manipulation of community taxonomic diversity data. PLoS Comput Biol. 2017;13(2):e1005404. Epub 2017/02/22. doi: 10.1371/journal.pcbi.1005404. PubMed PMID: 28222096; PubMed Central PMCID: PMCPMC5340466.

6. Gu Z, Eils R, Schlesner M. Complex heatmaps reveal patterns and correlations in multidimensional genomic data. Bioinformatics. 2016;32(18):2847-9. Epub 2016/05/22. doi: 10.1093/bioinformatics/btw313. PubMed PMID: 27207943.

7. Brown CT, Irber L. sourmash: a library for MinHash sketching of DNA. J Open Source Software. 2016;1(5):27.
